# Postprandial Glycemic Control With Automated Insulin Delivery With Control‐IQ Technology: The Impact of Meal Composition

**DOI:** 10.1111/dom.71135

**Published:** 2026-07-20

**Authors:** Marco Marigliano, Claudia Piona, Elisa Morotti, Maddalena Macedoni, Parto Kharazizadeh, Valentina Mancioppi, Francesca Tomasselli, Mara Tommasi, Claudio Maffeis

**Affiliations:** ^1^ Section of Pediatric Diabetes and Metabolism, Department of Surgery, Dentistry, Pediatrics, and Gynecology University of Verona Verona Italy

**Keywords:** adolescents, automated insulin delivery, control‐IQ, high‐fat meal, high‐protein meal, postprandial glucose, time in range, type 1 diabetes

## Abstract

**Aims:**

To evaluate the efficiency of the AID system with Control‐IQ technology and carbohydrate counting (CHC)–based premeal bolus to control postprandial glucose (PPG) after mixed meals with different macronutrient compositions in adolescents with type 1 diabetes (T1D).

**Material and Methods:**

In this prospective crossover study, 25 adolescents with T1D using an automated insulin delivery (AID—Tandem Tslim X2 with Control‐IQ) consumed three iso‐caloric meals (745 kcal) on separate days: a standard meal (56.5% carbohydrate), a high‐fat (HF) meal (45.9% fat), and a high‐protein (HP) meal (24.4% protein). A CHC‐based bolus was administered 15 min before each meal. Continuous glucose monitoring metrics and AID insulin delivery were assessed over 6 h. The primary outcome was 6‐h time in range (TIR, 70–180 mg/dL).

**Results:**

Six‐hour TIR was significantly lower after HF (65.2%) and HP meals (49.9%) compared with the standard meal (84.3%) (*p* < 0.01 and *p* < 0.001, respectively). Postprandial glucose exposure (AUC above 140 and 180 mg/dL) was significantly higher after HF and HP meals at both 3 and 6 h. The AID system delivered significantly more insulin than the pre‐programmed basal for HF (+26% at 6 h, *p* < 0.05) and HP meals (+55% at 6 h, *p* < 0.001), without increasing hypoglycemia.

**Conclusions:**

Despite a substantial autonomous increase in insulin delivery, AID systems using Control‐IQ technology and a standard CHC‐based pre‐meal bolus have not achieved adequate postprandial glycemic control after HF and HP meals. Proactively timed, meal‐specific insulin strategies are required to improve postprandial outcomes after nutritionally complex meals.

## Highlights

- Why did we undertake this study?
  ○ Postprandial glycemic control after high‐fat and high‐protein meals remains challenging in adolescents with type 1 diabetes using automated insulin delivery (AID) systems.
- What question did we want to answer?
  ○ We evaluated whether carbohydrate–counting–based bolusing with Control‐IQ technology adequately controls postprandial glucose after meals with different macronutrient compositions.
- What did we find?
  ○ High‐fat and high‐protein meals significantly reduced 6‐h time in range and increased glucose exposure versus a standard meal, despite substantial autonomous insulin delivery by the AID system.
- What are the implications?
  ○ Carbohydrate counting alone may be insufficient for complex meals in AID users. Meal‐specific insulin strategies and macronutrient‐responsive algorithms may improve postprandial glycemic outcomes.

## 1 Introduction

Achieving optimal glycemic control in children and adolescents with Type 1 Diabetes (T1D) remains a major clinical challenge. The adoption of Continuous Glucose Monitoring (CGM) and Automated Insulin Delivery (AID) systems has improved overall glycemic outcomes, including time in range (TIR), glycemic variability, and hypoglycemia rates, in paediatric populations [1, 2]. AID systems have demonstrated meaningful improvements in HbA1c and TIR in randomised controlled trials and real‐world studies [3, 4, 5, 6]. However, the postprandial period remains one of the most challenging phases of diabetes management, even for users of state‐of‐the‐art hybrid closed‐loop systems [7, 8].

The Tandem Tslim X2 with Control‐IQ technology is among the most widely used AID systems in paediatric patients. Its efficacy in improving TIR and reducing hypoglycemia has been demonstrated in randomised trials and real‐world studies, including in children as young as 6 years [9, 10, 11]. The Control‐IQ algorithm employs a model predictive controller that targets a glucose reference range of approximately 112.5–160 mg/dL. It modulates basal insulin delivery continuously and delivers automatic correction boluses when projected 30‐min glucose values are predicted to exceed 180 mg/dL. However, its performance in the specific context of nutritionally complex meals has received limited attention.

Meal composition is a principal determinant of postprandial glycemia. While carbohydrate content has historically driven mealtime insulin dosing through carbohydrate counting (CHC), dietary fat and protein exert independent, clinically important effects that are not captured by CHC alone [12, 13]. High dietary fat delays gastric emptying and promotes insulin resistance, producing prolonged hyperglycemia beyond the standard 2‐ to 3‐h postprandial window [14]. High dietary protein stimulates glucagon secretion and hepatic glucose production, generating a secondary delayed hyperglycemic phase [15]. The combined effect produces a biphasic, often unpredictable PPG profile that is poorly addressed by a single CHC‐based pre‐meal bolus. Moreover, postprandial glucose excursions exert independent harmful effects beyond their contribution to time‐averaged glycemic metrics: even transient hyperglycemia activates oxidative and inflammatory pathways that promote endothelial dysfunction and are associated with long‐term microvascular and cardiovascular complications. This makes postprandial glycemic control a clinically important target distinct from overall TIR optimization.

The clinical relevance of these effects is heightened in the real‐world dietary patterns of children and adolescents, who frequently consume meals that deviate considerably from nutritionally balanced compositions, including ultra‐processed fast food, pizza, and other high‐fat or high‐protein options [16]. Understanding how AID systems respond to such meals is therefore not only scientifically informative but directly relevant to clinical practise and patient counselling.

We previously demonstrated in adolescents with T1D using non‐automated insulin pumps that a second corrective bolus administered 3 h after HF and HP meals—calculated as 30% and 60% of the CHC bolus, respectively—significantly reduced PPG AUC and increased TIR without increasing hypoglycemia risk [17]. That manually administered bolus was deliberately timed and sized to anticipate the delayed hyperglycemic impact of dietary fat and protein, whereas the Control‐IQ algorithm's automatic correction boluses are generated by a predictive algorithm whose glucose forecast is derived solely from recent glucose trends and insulin dosing history, without incorporating meal type or macronutrient composition; corrections are triggered only once glucose is predicted to exceed 180 mg/dL and are capped at 60% of the calculated correction dose, with a maximum of one bolus per hour. Based on these design constraints, the autonomous response would be expected, a priori, to provide at most a partial and delayed compensation, rather than full normalisation of postprandial glycemia. Whether contemporary AID systems can autonomously approximate the magnitude of benefit previously achieved with this proactively timed manual strategy, and to what extent a clinically meaningful gap persists relative to glycemic targets, remains incompletely characterised—a question of direct clinical relevance independent of the anticipated direction of the result.

The present study evaluated whether a single pre‐meal CHC bolus administered 15 min before a real‐life meal controls the 6‐h PPG profile after three iso‐caloric test meals with distinct macronutrient compositions (standard, HF, and HP) in adolescents with T1D using Control‐IQ. A secondary aim was to quantify the autonomous AID insulin augmentation required to partially compensate for the glycemic impact of HF and HP meals. Even though the capped nature of automatic correction boluses made an incomplete compensatory response the most plausible a priori expectation, precise quantification of its magnitude, time course, and safety under real‐life single‐bolus dosing is clinically necessary to benchmark the residual gap relative to glycemic targets and to inform the design of future meal‐specific correction strategies.

## 2 Materials and Methods

### 2.1 Study Design

This was an exploratory, mono‐center, non‐blinded, crossover, repeated‐measures study conducted at the Regional Center for Paediatric Diabetes, University of Verona, Italy. The study was approved by the local ethics committee (cod. 3773CESC) and conducted in accordance with the Declaration of Helsinki. Written informed consent was obtained from all participants and/or their parents or legal guardians prior to any study procedure.

### 2.2 Subjects

Inclusion criteria were age 6–18 years; T1D diagnosis for at least 12 months; current use of the Tandem Tslim X2 with Control‐IQ technology in combination with Dexcom G6 or G7 CGM for at least 3 months; HbA1c < 9.0% (75 mmol/mol); and ability to consume all three test meals. All participants had received standard carbohydrate counting education as part of their routine diabetes care per ISPAD guidelines; no additional study‐specific training was provided. Exclusion criteria included celiac disease or other gastrointestinal disorders affecting nutrient absorption, relevant food allergies or intolerances, gastroparesis, additional chronic conditions affecting glucose metabolism, or any significant change in diabetes therapy within the preceding 3 months. The participants' BMI‐SDS was calculated according to WHO charts [18] and followed a standard nutrition education program based on ISPAD guidelines [19].

### 2.3 Intervention

During the 48 h before each test day, patients were instructed not to follow a hypocaloric diet or engage in intense physical activity; compliance was assessed by self‐report. Continuous subcutaneous insulin infusion was administered by patients' AID system (Tandem Tslim X2, Tandem Diabetes Care, San Diego, CA, USA) using rapid‐acting insulin (Humalog, Eli Lilly, Indianapolis, IL, USA), and interstitial glucose was measured continuously with a CGM (Dexcom G6/G7, Dexcom, San Diego, CA, USA).

Each enrolled participant consumed three iso‐caloric test meals 745 kcal each with different nutrient composition: Meal A [standard meal (protein; lipid; carbohydrate; percent of meal energy: 14.0%; 29.5%; 56.5%)], Meal B [high‐fat (HF) meal (16.1%; 45.9%; 38.0%)], and Meal C [high‐protein (HP) meal (24.4%; 30.0%; 45.6%)] (see Table S1). The three test meals were consumed on non‐consecutive days within a 4‐week window, with a minimum 48‐h interval between visits to minimise carryover effects. An iso‐caloric design was chosen to replicate real‐world conditions in which adolescents consume meals of similar energy content but divergent macronutrient profiles. A single insulin bolus was calculated using the individual CHC method (based on the participant's established insulin‐to‐carbohydrate ratio) and administered via the AID system 15 min before each meal. A standard single pre‐meal bolus without extended delivery was deliberately employed to reflect common clinical practise and to characterise the baseline response of the AID system under these conditions. Pre‐meal bolus administration 15 min before meal start was selected to reflect standard clinical practise and is consistent with recommendations for rapid‐acting insulin analog administration in paediatric patients. No manual correction boluses were administered during the 6‐h observation period; any additional insulin delivered was exclusively the result of autonomous AID system action. All test meals were administered at lunchtime (12:00–13:00). A pre‐meal sensor glucose of 70–120 mg/dL was required before bolus administration; participants arrived without active insulin on board from prior correction boluses. CGM data were continuously collected over the 6‐h postprandial observation window, starting at the time of meal initiation (first bite).

### 2.4 Outcomes

The primary outcome was 6‐h postprandial Time in Range (PPG‐TIR, 70–180 mg/dL). Secondary outcomes included: mean glucose, coefficient of variation (CV), time in tight range (TITR, 70–140 mg/dL), time above range level 1 (TAR‐L1, 180–250 mg/dL) and level 2 (TAR‐L2, > 250 mg/dL), time below range level 1 (TBR‐L1, 54–69 mg/dL) and level 2 (TBR‐L2, < 54 mg/dL), PPG AUC above 140 mg/dL and 180 mg/dL, PPG time spent over 140 and 180 mg/dL. Moreover, the differences (expressed as absolute IU and percentages) between total automated insulin delivered by the AID system (automated basal + correction boluses) and the programmed basal insulin over the 3‐ and 6‐h PPG windows were evaluated.

### 2.5 Statistical Analysis

The Kolmogorov–Smirnov test was used to assess the normality of the variables. Descriptive statistics are reported as the mean (standard deviation, SD) for normally distributed continuous variables or as median (interquartile range, IQR) for not normally distributed ones. Categorical data are presented as absolute frequencies and percent values. Sex differences in baseline characteristics were assessed by an independent‐samples *t*‐test or the Mann–Whitney *U* test. Comparison of AID‐delivered insulin versus the programmed pre‐meal bolus was performed using paired Student's *t*‐test or the Wilcoxon rank test. Differences in PPG metrics across the three meals were assessed by repeated‐measures ANOVA with Bonferroni post hoc correction. Statistical significance was set at *p* < 0.05. Statistical analysis was performed using the Jamovi project (2025) [jamovi (Version 2.6); Retrieved from https://www.jamovi.org] and GraphPad Prism version 10 (GraphPad Software, San Diego, CA).

This was an exploratory study, and the sample size was not determined by a formal a priori power calculation. Post hoc power analysis confirmed the observed effect size (Cohen's *f* = 1.06, large) yielded > 99% retrospective power for the overall ANOVA and 94%–99% power for pairwise comparisons at *n* = 25, with a minimum detectable TIR difference of approximately 10.9 percentage points at 80% power, at or below the internationally recognised minimum clinically important difference.

## 3 Results

### 3.1 Participant Characteristics

A total of 25 adolescents with T1D were enrolled (52% males; mean age 13.8 ± 2.1 years). Baseline characteristics are summarised in the Table S2. Mean T1D duration was 9.0 ± 3.3 years, and participants had been using the Control‐IQ system for a mean of 3.1 ± 1.5 years. Mean HbA1c was 6.97% ± 0.60% (52.7 ± 6.4 mmol/mol), with no significant sex differences in HbA1c or BMI. In Table 1, the overall CGM‐derived metrics show a mean glucose of 157.0 ± 16.6 mg/dL and a TIR of 69.8% ± 9.4%. Mean glucose and GMI were significantly higher in males than in females (*p* = 0.039), whereas TIR was lower in males (*p* = 0.021). All 25 participants completed enrollment and all three meal challenges with full CGM data and were included in the primary PPG analysis.

**TABLE 1:** CGM glucometrics at the baseline of the study cohort.

	Total (*n* = 25)	Males (*n* = 13)	Females (*n* = 12)	*p*
CGM active (%)	94.5 ± 4.1	94.0 ± 4.4	95.0 ± 3.9	0.620
Mean glucose (mg/dL)	157.0 ± 16.6	165.4 ± 17.5	148.7 ± 11.2	0.039*
CV (%)	35.0 ± 5.2	37.0 ± 4.2	33.0 ± 5.6	0.126
TBR L2 (< 54 mg/dL) (median, IQR)	0.22 (0.60)	0.28 (0.59)	0.20 (0.60)	0.958
TBR L1 (54–69 mg/dL)	1.65 ± 1.30	1.50 ± 1.29	1.80 ± 1.31	0.658
TIR (70–180 mg/dL)	69.8 ± 9.4	64.7 ± 8.7	75.1 ± 7.1	0.021*
TITR (70–140 mg/dL)	42.2 ± 11.1	38.1 ± 11.6	46.2 ± 9.7	0.153
TAR L1 (180–250 mg/dL)	20.6 ± 6.4	22.6 ± 6.9	18.6 ± 5.6	0.224
TAR L2 (> 250 mg/dL)	7.42 ± 5.6	10.7 ± 5.8	4.1 ± 2.9	0.012*
GMI (%)	7.07 ± 0.40	7.27 ± 0.41	6.87 ± 0.39	0.039*
GMI (mmol/mol)	53.7 ± 4.3	55.9 ± 4.6	51.5 ± 2.9	0.039*

*Note:* Data presented as mean ± SD or median and IQR.

Abbreviations: CGM, continuous glucose monitoring; GMI, glucose management indicator; TAR, time above range; TBR, time below range; TIR, time in range.

* *p* < 0.05.

### 3.2 Postprandial Glycemic Metrics by Meal Type

Postprandial CGM metrics over 3 and 6 h for each meal are presented in Table 2.

**TABLE 2:** Postprandial CGM metrics (3 h PPG vs. 6 h PPG) after three test meals [Meal A (Standard—Pasta); Meal B (High‐Fat—Cheeseburger); Meal C (High‐Protein—Pizza)].

	Meal A		*p*	Meal B		*p*	Meal C		*p*
	CHO (g)	Bolus (IU)		CHO (g)	Bolus (IU)		CHO (g)	Bolus (IU)	
	112.0	7.4 ± 3.3		71.0	5.9 ± 2.6		85.0	6.1 ± 3.1	
	3 h PPG	6 h PPG		3 h PPG	6 h PPG		3 h PPG	6 h PPG	
TBR L2 (< 54 mg/dL) (%) (median, IQR)	0.0 (0.0)	0.0 (0.0)	1.000	0.0 (0.0)	0.0 (0.0)	1.000	0.0 (0.0)	0.0 (0.0)	1.000
TBR L1 (54–69 mg/dL) (%) (median, IQR)	0.0 (8.1)	0.0 (4.5)	0.352	0.0 (0.0)	0.0 (2.1)	0.787	0.0 (2.0)	0.0 (4.1)	0.416
TBR (< 70 mg/dL) (%) (median, IQR)	0.0 (8.1)	0.0 (5.8)	0.553	0.0 (0.0)	0.0 (2.0)	1.000	0.0 (2.0)	0.0 (4.1)	0.588
TIR 70–180 mg/dL (%)	78.2 ± 26.0	84.3 ± 16.2	0.076	72.8 ± 20.6	65.2 ± 14.6	0.163	64.0 ± 23.7	49.9 ± 23.9	0.001*
TITR 70–140 mg/dL (%)	58.4 ± 32.7	54.5 ± 29.6	0.427	44.4 ± 27.5	34.9 ± 18.8	0.110	38.5 ± 23.4	26.0 ± 17.2	0.002*
TAR > 180 mg/dL (%) (median, IQR)	0.0 (38.5)	1.4 (22.9)	0.141	20.3 (23.0)	32.9 (24.0)	0.222	35.1 (44.6)	54.1 (37.0)	0.006*
TAR L1 180–250 mg/dL (%) (median, IQR)	0.0 (38.5)	1.4 (22.6)	0.141	18.9 (13.5)	24.0 (14.7)	0.244	17.6 (19.6)	30.8 (33.9)	0.007*
TAR L2 > 250 mg/dL (%) (median, IQR)	0.0 (0.0)	0.0 (0.0)	1.000	0.0 (2.7)	0.0 (6.2)	0.590	1.4 (16.2)	6.2 (28.8)	0.221
Mean glucose (mg/dL)	129 ± 36.9	131.0 ± 28.2	0.698	150.0 ± 27.3	158.0 ± 25.5	0.295	164.0 ± 42.6	184.0 ± 44.3	0.009*
CV (%)	19.4 ± 8.8	23.2 ± 8.0	0.071	25.7 ± 10.1	27.5 ± 9.5	0.320	28.5 ± 10.4	27.8 ± 10.1	0.740
PPG AUC > 140 mg/dL (mg/dL·min) (median, IQR)	920 (5452)	3286 (8084)	0.002*	3656 (3650)	11 010 (4996)	< 0.001*	5729 (7909)	18 614 (12329)	< 0.001*
PPG TIME > 140 mg/dL (min) (median, IQR)	57.5 (105)	145 (201)	0.002*	108 (71)	235 (75)	< 0.001*	110 (77.5)	253 (128)	< 0.001*
PPG AUC > 180 mg/dL (mg/dL·min) (median, IQR)	0.0 (1323)	16.3 (1436)	0.059	651 (1889)	3539 (4341)	0.002*	2319 (4545)	7653 (9989)	< 0.001*
PPG TIME > 180 mg/dL (min) (median, IQR)	0.0 (68.8)	7.5 (91.3)	0.059	42.5 (41.3)	123.0 (91.3)	0.002*	65.0 (83.8)	193 (135)	< 0.001*

*Note:* The *p*‐values in each meal column represent the within‐meal comparison between the 3‐h and 6‐h postprandial windows (paired analysis). Cross‐meal comparisons are reported in the main text.

Data presented as mean ± SD or median and IQR.

Abbreviations: AUC, area under the curve; CHO, carbohydrates; PPG, postprandial glucose; TAR, time above range; TBR, time below range; TIR, time in range; TITR, time in tight range.

* *p* < 0.05.

Across all three meals, median whole TBR remained below 4% and median TBR‐L2 below 1%, consistent with recommended safety thresholds. No hypoglycemic episode requiring treatment was observed. At 3 h, PPG‐TIR was 78.2%, 72.8%, and 64.0% for Meals A, B, and C, respectively; differences at 3 h did not reach statistical significance for TIR (*p* = NS), but PPG AUC > 140 mg/dL was already significantly higher for Meal C vs. Meal A (*p* = 0.031).

After Meal A (standard), mean PPG at 6 h was 131.0 ± 28.2 mg/dL, with a PPG‐TIR of 84.3% ± 16.2% and PPG‐TITR of 54.5% ± 29.6%, reflecting generally well‐controlled glycemia achievable with a CHC bolus alone.

Following Meal B (HF), mean PPG rose to 158.0 ± 25.5 mg/dL at 6 h. The 6‐h PPG‐TIR was significantly lower than after Meal A (65.2% ± 14.6% vs. 84.3% ± 16.2%; *p* < 0.01), as was TITR (34.9% ± 18.8% vs. 54.5% ± 29.6%; *p* < 0.05). The 6‐h PPG AUC > 140 mg/dL more than tripled compared to Meal A [11,010 (4996) vs. 3286 (8084) mg/dL·min; *p* < 0.01]; similarly, PPG AUC > 180 mg/dL was significantly elevated [3539 (4341) vs. 16.3 (1436) mg/dL·min; *p* < 0.01]. Moreover, the time spent over the 140 mg/dL and the 180 mg/dL thresholds is increased (approximately +90 additional minutes above 140 mg/dL and +116 additional minutes above 180 mg/dL).

**FIGURE 1:**
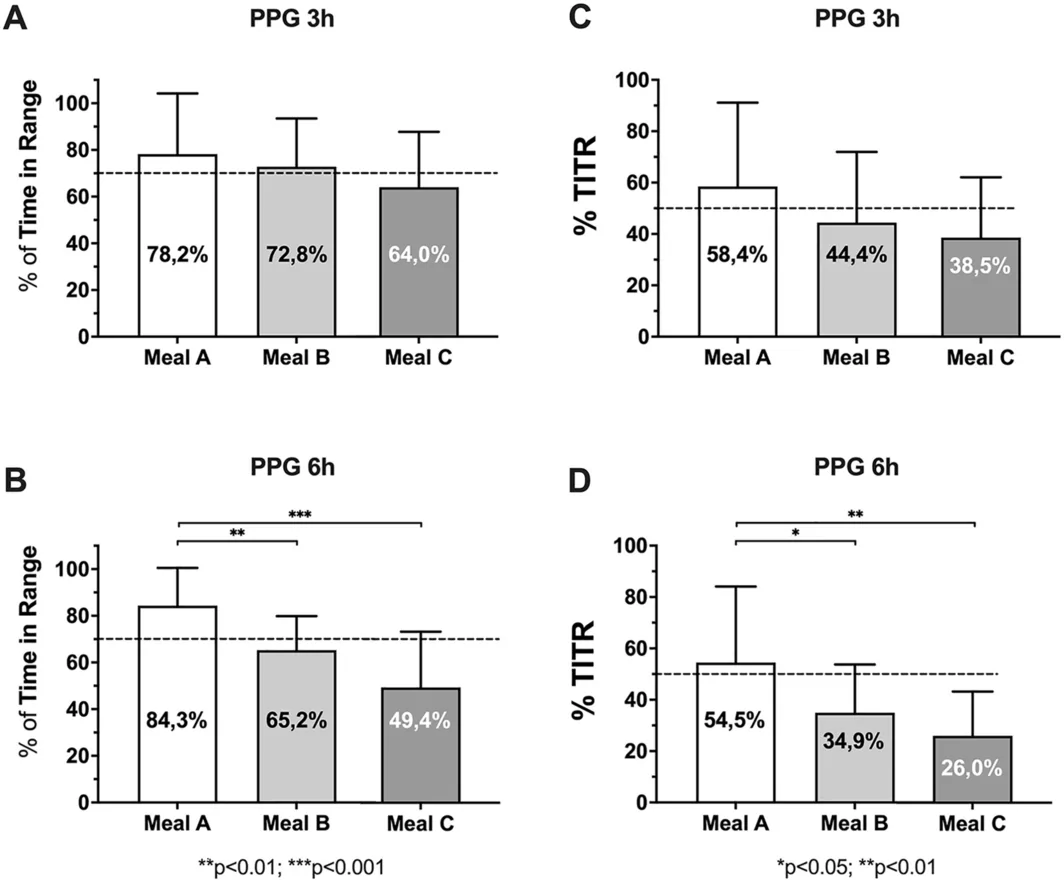
Postprandial time in range (PPG‐TIR, 70–180 mg/dL) and time in tight range (PPG‐TITR, 70–140 mg/dL) at 3 and 6 h after each test meal. Panel A and C: Three hours PPG‐TIR and PPG‐TITR comparisons (Meal A vs. Meal B vs. Meal C); Panel B and D: Six hours PPG‐TIR and PPG‐TITR comparisons (Meal A vs. Meal B vs. Meal C); the dashed line indicates the 70% TIR clinical target and the 50% TITR clinical target. White = Meal A (standard); Light Grey = Meal B (high‐fat); Dark Grey = Meal C (high‐protein). *p < 0.05; **p < 0.01; ***p < 0.001.

**FIGURE 2:**
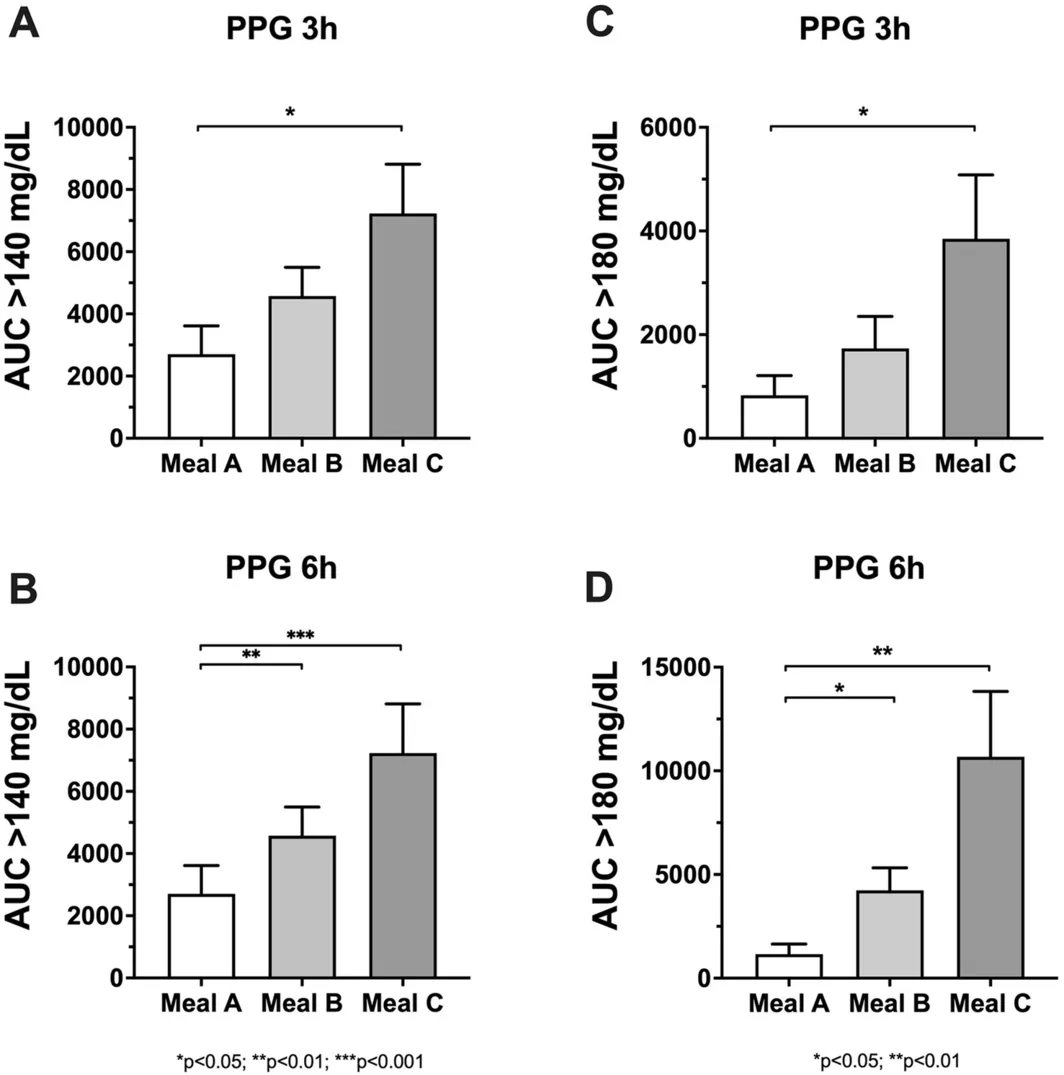
Postprandial AUC above 140 mg/dL and 180 mg/dL at 3 and 6 h after each test meal. Panel A and C: Three hours AUC > 140 mg/dL and AUC > 180 mg/dL comparisons (Meal A vs. Meal B vs. Meal C); Panel B and D: Six AUC > 140 mg/dL and AUC > 180 mg/dL comparisons (Meal A vs. Meal B vs. Meal C). White = Meal A (standard); Light Grey = Meal B (high‐fat); Dark Grey = Meal C (high‐protein). *p < 0.05; **p < 0.01; ***p < 0.001.

Following Meal C (HP), the most pronounced PPG excursions were observed. Mean PPG reached 184.0 ± 44.3 mg/dL at 6 h. The 6‐h PPG‐TIR fell to 49.9% ± 23.9%, significantly below both Meal A (*p* < 0.001) and nominally below Meal B. The 6‐h PPG AUC > 140 mg/dL was 18,614 (12,328) mg/dL·min, and PPG AUC > 180 mg/dL was 7653 (9989) mg/dL·min, both significantly higher than Meal A (*p* < 0.01). The time spent above the two thresholds (140 and 180 mg/dL) was increased by +108 min and +186 min, respectively, vs. Meal A. Critically, no significant differences in TBR‐L1 or TBR‐L2 were observed across the three meals at either 3 or 6 h. PPG‐TIR and PPG‐TITR across meals at 3 and 6 h are shown in Figure 1. Figure 2 shows PPG AUC > 140 and > 180 mg/dL after the three meals.

### 3.3 Autonomous AID Insulin Delivery vs. Pre‐Programmed Basal

**FIGURE 3:**
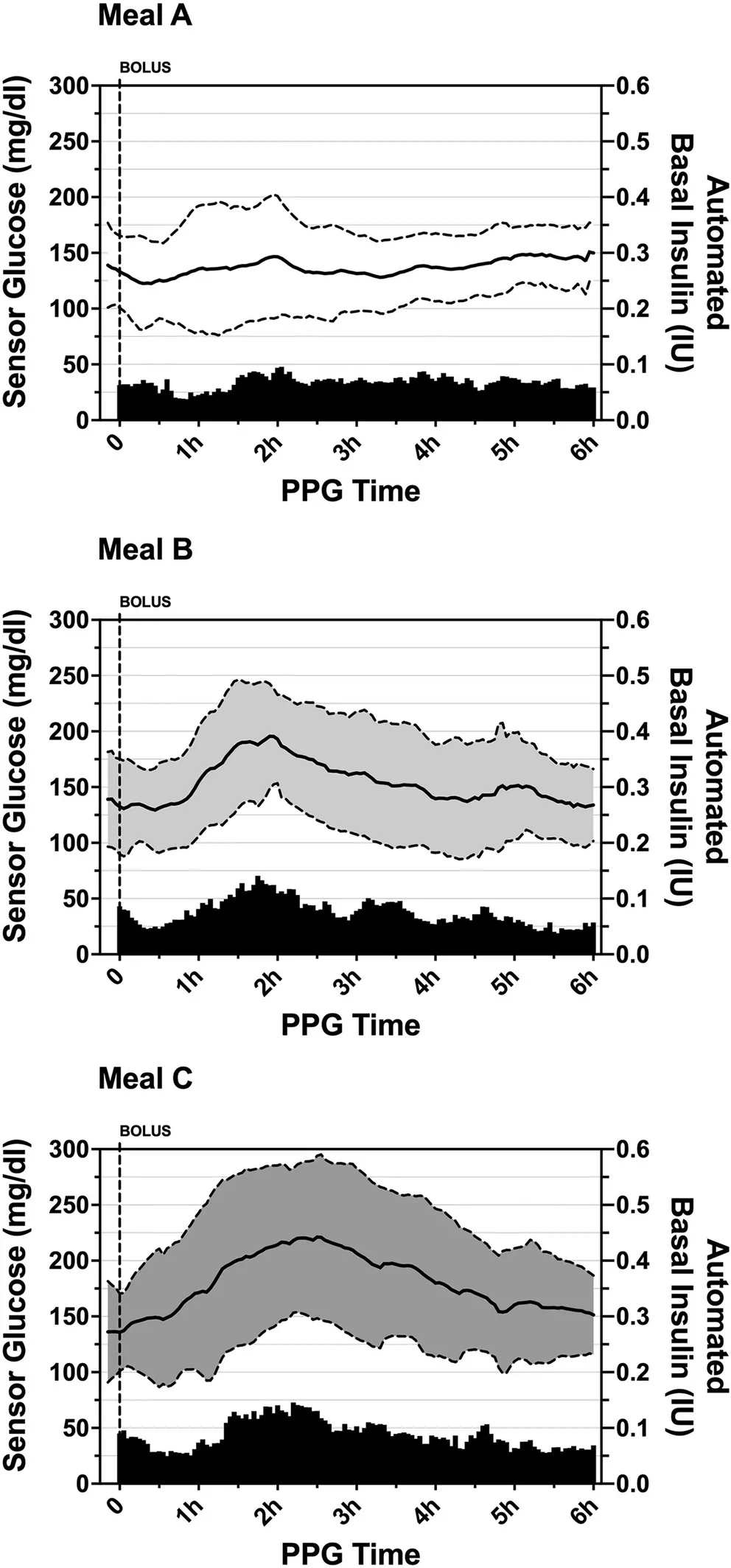
Postprandial glucose levels (left *Y* axis) and automated basal insulin delivery (right *Y* axis) in the 6 h of observation after each test meal (Meal A, Meal B, and Meal C).

The glucose profiles and automated basal insulin delivery were markedly meal‐dependent (Figure 3). After Meal A, the AID system delivered less total insulin (automated basal insulin + correction boluses) than the pre‐programmed basal insulin at both 3 h (2.46 ± 1.06 IU vs. 3.15 ± 1.23 IU; *p* = 0.065) and 6 h (5.33 ± 2.28 IU vs. 6.14 ± 2.12 IU; *p* = 0.260), equal to −0.69 IU (−21%) and −0.81 IU (−6%), consistent with adequate early glycemic control by the CHC bolus alone, preventing hypoglycemic events.

After Meal B, the AID system delivered approximately the same amount of insulin as pre‐programmed at 3 h (3.29 ± 1.60 IU vs. 3.18 ± 1.29 IU; *p* = 0.736) and significantly more at 6 h (7.76 ± 2.99 IU vs. 6.17 ± 2.18 IU; *p* < 0.05), equal to +1.59 IU (+26%). After Meal C, the most pronounced autonomous augmentation was observed: approximately +0.49 IU (+15%) above pre‐programmed at 3 h (3.70 ± 1.81 IU vs. 3.21 ± 1.28 IU; *p* = 0.149), rising to +3.36 IU (almost +55%) above pre‐programmed at 6 h (9.54 ± 2.54 IU vs. 6.18 ± 2.19 IU; *p* < 0.001). Despite this substantial autonomous compensatory insulin delivery, glycemic targets were not achieved after either HF or HP meals. No clinically significant hypoglycemia was observed across any of the three meals.

## 4 Discussion

AID systems represent the most effective therapeutic option for glycemic control in youths with T1D and are strongly recommended by international guidelines across all age groups [20], yet cannot guarantee optimal postprandial glycemic control after all meal types.

The present study showed that, in a cohort of adolescents with T1D using the Tandem Tslim X2 with Control‐IQ AID, a single pre‐meal insulin bolus calculated solely by CHC achieved optimal postprandial glycemic control with a standard, nutrient‐balanced meal, but not with high‐fat or high‐protein meals. Despite the AID system autonomously delivering substantially more insulin than pre‐programmed—up to 26% more for the HF meal and up to 55% more for the HP meal at 6 h—the 6‐h PPG‐TIR after HF and HP meals was significantly lower than after the nutritionally balanced standard meal, and PPG AUC above both 140 and 180 mg/dL was markedly elevated. Crucially, this occurred without any increase in hypoglycemia, highlighting that the limitation lies not in insulin function but in the fundamental challenge of delivering insulin with sufficient speed and volume to match the delayed and prolonged hyperglycemic impact of dietary fat and protein.

These findings extend our prior work in a similar paediatric population using non‐automated insulin pump therapy, in which a corrective bolus administered 3 h after HF and HP meals—calculated as 30% and 60% of the original CHC bolus, respectively—significantly improved PPG outcomes without increasing hypoglycemia [17]. The convergence between empirically derived correction bolus percentages and autonomous AID augmentation observed here suggests that fat‐ and protein‐driven metabolic demands follow predictable kinetic patterns, yet even with this augmentation, a meaningful gap persists between observed TIR and guideline targets such as TITR, highlighting postprandial control after nutritionally complex meals as a critical remaining opportunity to improve outcomes in young people using AID.

The differential time courses of glycemic impact observed for HF versus HP meals are consistent with established mechanistic understanding. High dietary fat primarily slows gastric emptying through cholecystokinin‐mediated pyloric contraction and duodenal lipid sensing, attenuating early postprandial glucose absorption while subsequently promoting prolonged hyperglycemia via increased non‐esterified fatty acid flux, reduced peripheral insulin sensitivity, and impaired glucose uptake in skeletal muscle and adipose tissue [14, 21]. This explains why differences between HF and standard meals were most pronounced at 6 h: at 3 h, the reduced CHO content of Meal B (71 g vs. 112 g) partially compensated for fat‐driven delayed hyperglycemia, but by 6 h, fat‐mediated insulin resistance became dominant. High dietary protein, by contrast, exerts glucagonotropic effects independent of gastric transit, stimulating alpha‐cell glucagon secretion and hepatic glucose production that begins within 2–3 h of ingestion and persists for up to 5–6 h [15, 22].

The performance characteristics of Control‐IQ under these meal conditions deserve specific examination. Control‐IQ's predictive controller targets a glucose range of 112.5–160 mg/dL, increases basal delivery when projected 30‐min glucose exceeds 160 mg/dL, and triggers automatic correction boluses above 180 mg/dL—capped at 1 per hour to reduce hypoglycemia risk [11]. Critically, this glucose forecast is derived exclusively from CGM trends and recent insulin dosing history; meal type and macronutrient composition are not inputs to the prediction model, even though meal composition is the principal determinant of the postprandial glucose excursions described here. This is precisely why the system's automated insulin delivery, however substantial in magnitude, is insufficient to adequately manage postprandial glucose excursions after nutritionally complex meals. While well‐suited to managing gradual glycemic fluctuations, this design may be inherently constrained in delivering insulin quickly and in sufficient volume to match the steep, sustained elevations characteristic of HP meals. Our data suggest that even 55% additional insulin over 6 h is insufficient to overcome the combined gluconeogenic and glucagonotropic burden of 45.5 g of protein, consistent with reports from other authors demonstrating suboptimal PPG control in AID users consuming high‐protein or mixed macronutrient meals [23, 24]. Prior randomised trials of Control‐IQ [9, 11] and real‐world cohorts reporting mean TIR of 65%–70% [25] relied on TIR averaged over weeks as the primary outcome and did not characterise postprandial performance after nutritionally diverse meals. Our cohort's overall 2‐week TIR of approximately 70% is consistent with these benchmarks, confirming that the population was representative and well‐engaged with the technology. Yet meal‐specific PPG‐TIR for the HP meal fell to below 50%, a dramatic within‐person deterioration that aggregate TIR metrics alone would not reveal.

Several mealtime strategies have been proposed to improve PPG control after nutritionally complex meals. The currently validated correct approach for HF and HP meals is the manually administered second corrective bolus we previously described [17]: a bolus given approximately 3 h after the meal, sized at 30% of the original CHC dose for HF meals and 60% for HP meals, which significantly reduced PPG AUC and increased TIR without increasing hypoglycemia risk. This proactive, appropriately timed strategy directly addresses the limitation identified in the present study: the AID system's automatic corrections are generated by a predictive algorithm that does not incorporate meal type into its glucose forecast, are capped at 60% of the calculated correction dose, and are limited to one per hour, and therefore cannot replicate this benefit. Extended/dual‐wave boluses and meal‐announcement or manual bolus override strategies have shown variable benefit in closed‐loop settings [23, 26, 27]. Incorporating dietary fat and protein estimates into mealtime dosing—through simplified correction factors or AI‐based meal recognition algorithms—represents an active area of development [28]. Our data directly support this practical approach: the magnitude of AID augmentation required for HF and HP meals is predictable and could, in principle, be pre‐specified as a meal‐specific correction factor alongside the standard CHC bolus.

This study has several potential limitations. The sample size of 25 participants with complete meal data is modest for a crossover design, and findings require replication in larger, more diverse cohorts. They had relatively well‐controlled glycemia (mean HbA1c 7.0%; 53 mmol/mol and TIR about 70%), which may limit generalizability to populations with suboptimal baseline control. The study was conducted in a single center in Italy, and generalizability to populations with different dietary patterns, body weight distributions, or AID use durations may be limited. The iso‐caloric design of standardised meals (not formally randomised) may not fully reflect the variability in real‐world meal composition and size. Nevertheless, this replicates real‐world conditions in which adolescents consume meals of similar energy content but divergent macronutrient profiles, contrasting with a fixed‐carbohydrate design that would isolate fat/protein effects but provide higher energy than the reference meal and lack ecological validity. Moreover, the test meals featured macronutrient profiles typical of adolescent dietary choices but were not representative of all HF or HP foods; other foods with different fatty acid compositions may have different effects on gastric emptying and glucose response [29]. The Control‐IQ system was used in standard mode throughout; Sleep Mode performance, which applies a lower glucose target, was not specifically evaluated. Physical activity restriction was assessed solely by self‐report; residual effects of prior exercise on insulin sensitivity cannot be entirely excluded and may contribute to variability in postprandial glycemic responses. The 6‐h observation window may not fully capture the glycemic tail of high‐fat meals. Finally, we did not separately quantify the contributions of increased basal delivery and the correction bolus to the total AID insulin augmentation, which would provide additional mechanistic insight into how the algorithm responds to each meal type.

It is also worth noting that Meal B (HF) and Meal C (HP) are not extreme dietary challenges. Dietary surveys across European countries consistently document mean fat intakes of 35%–40% of total energy and protein intakes of 15%–20% in children and adolescents, frequently exceeding age‐specific recommendations [30, 31], while fibre consumption remains substantially below guidelines [32]. The meals tested here, therefore, reflect habitual adolescent eating patterns, reinforcing the clinical relevance of our findings for everyday diabetes management.

Strengths of the study include the use of real‐life meals representative of typical adolescent dietary choices, controlled laboratory conditions with standardised meal timing and continuous CGM monitoring, novel simultaneous characterisation of glycemic outcomes and insulin delivery dynamics, and direct comparison of autonomous AID compensation with previously established manual correction bolus requirements. The consistent finding of no hypoglycemia across all three meals—despite substantially greater insulin delivery for HF and HP meals—is an important safety reassurance.

In conclusion, high‐fat and high‐protein meals continue to challenge postprandial glycemic control in adolescents with T1D, even with Control‐IQ AID technology. Carbohydrate counting alone is insufficient to inform mealtime dosing for HF and HP meals, and the AID system's autonomous compensation remains lacking to achieve glycemic outcomes comparable to those after a nutritionally balanced meal. Clinicians should counsel patients and families about the glycemic effects of dietary fat and protein and should consider a proactive manual correction bolus as an adjunct to standard CHC bolusing since the AID system's automatic corrections, generated by a predictive algorithm that is constrained by dose caps and hourly limits, cannot replicate this benefit. Hopefully, the use of new ultra‐rapid‐acting insulins and the design of specific macronutrient‐responsive AID algorithms will help to improve postprandial control.

## Author Contributions

M.M. and C.M. were involved in the conception, design, and conduct of the study, as well as in the analysis and interpretation of the results. M.M. wrote the first draft of the manuscript, and C.P., E.M., M.Mac., P.K., V.M., F.T., M.T., and C.M. edited, reviewed, and approved the final version of the manuscript. C.M. is the guarantor of this work and, as such, had full access to all the data in the study and takes responsibility for the integrity of the data and the accuracy of the data analysis. The datasets generated during and/or analysed in the current study are available from the corresponding author upon reasonable request.

## Funding

The authors have nothing to report.

## Conflicts of Interest

M.M. reports a relationship with Ypsomed Diabetes SL that includes consulting or advisory, speaking and lecture fees, and travel reimbursement; Movi S.p.A. that includes consulting or advisory; Theras Lifetech Srl that includes speaking and lecture fees; Abbott Italy that includes speaking and lecture fees; Medtronic MiniMed Inc. that includes consulting or advisory and speaking and lecture fees. C.P. reports a relationship with Sanofi that includes consulting or advisory. E.M. reports a relationship with Movi S.p.A. that includes travel reimbursement. C.M. reports a relationship with Sanofi that includes consulting or advisory, speaking and lecture fees, and travel reimbursement; Abbott that includes board membership, consulting or advisory, speaking and lecture fees, and travel reimbursement; Medtronic MiniMed Inc. that includes consulting or advisory and speaking and lecture fees; Thereas that includes speaking and lecture fees and travel reimbursement. The other authors declare no conflicts of interest.

## Supporting information


**Table S1:** Nutrient composition of test meals.
**Table S2:** Anthropometric, glycemic control, and insulin treatment baseline characteristics of the study cohort.

## Data Availability

The data that support the findings of this study are available from the corresponding author upon reasonable request.
