# Supplementary material for: Postprandial Glycemic Control With Automated Insulin Delivery With Control‐IQ Technology: The Impact of Meal Composition
Source: Diabetes Obes Metab. 2026 Jul 20;28(10):9178–87. doi: 10.1111/dom.71135 (PMC13538753; doi:10.1111/dom.71135)
Supplement: Supplementary file 1 [file DOM-28-9178-s001.docx]

**Supplementary Table 1**

Nutrient composition of test meals.

| **MEALS** | **Energy**  (kcal) | **PROTEIN g**  (% energy) | **LIPIDS g**  (% energy) | **CHO g**  (% energy) | **FIBER**  (g) |
| --- | --- | --- | --- | --- | --- |
| **Meal A - Standard**  (Pasta with tomato sauce and parmesan cheese, Ham, salad with olive oil, bread) | 745 | 26.0 (14.0) | 24.5 (29.5) | 112.0 (56.5) | 6.1 |
| **Meal B – High Fat (HF)**  (Fast-food meal, cheeseburger with potato chips) | 745 | 30.0 (16.1) | 38.0 (45.9) | 71.0 (38.0) | 5.0 |
| **Meal C – High Protein (HP)**  (Pizza with tomato sauce, mozzarella cheese and ham with mushrooms) | 745 | 45.5 (24.4) | 24.8 (30.0) | 85.0 (45.6) | 5.5 |

**Supplementary Table 2**

Anthropometric, glycemic control, and insulin treatment baseline characteristics of the study cohort

|  | Total (n=25) | Males (n=13) | Females (n=12) | p |
| --- | --- | --- | --- | --- |
| Age (years) *(range 10-18y)* | 13.8 ± 2.1 | 13.8 ± 2.4 | 13.9 ± 1.9 | 0.930 |
| T1D duration (years) | 9.0 ± 3.3 | 8.7 ± 3.4 | 9.2 ± 3.3 | 0.737 |
| Control-IQ use (years) | 3.1 ± 1.5 | 3.0 ± 1.4 | 3.3 ± 1.6 | 0.594 |
| Weight (kg) | 50.9 ± 11.1 | 50.4 ± 13.4 | 51.7 ± 8.5 | 0.793 |
| Height (cm) | 160.0 ± 13.0 | 160.6 ± 16.3 | 159.4 ± 8.7 | 0.825 |
| BMI (kg/m²) | 19.6 ± 2.2 | 19.1 ± 2.0 | 20.2 ± 2.4 | 0.212 |
| BMI-SDS | 0.14 ± 0.70 | 0.04 ± 0.55 | 0.25 ± 0.83 | 0.454 |
| HbA1c (%) | 6.97 ± 0.60 | 7.07 ± 0.50 | 6.86 ± 0.60 | 0.380 |
| HbA1c (mmol/mol) | 52.7 ± 6.4 | 53.8 ± 5.8 | 51.5 ± 6.9 | 0.384 |
| Total Insulin (IU/d) | 43.6 ± 12.3 | 44.8 ± 14.1 | 42.3 ± 10.6 | 0.634 |
| Basal Insulin (IU/d) | 20.4 ± 7.0 | 21.3 ± 8.5 | 19.5 ± 5.3 | 0.530 |
| Prandial Insulin (IU/d) | 23.2 ± 9.1 | 23.5 ± 9.6 | 22.9 ± 8.8 | 0.872 |
| Total Insulin/BW (IU/kg/d) | 0.85 ± 0.15 | 0.88 ± 0.15 | 0.82 ± 0.15 | 0.285 |
| Time in CIQ (%) | 94.2 ± 3.9 | 93.4 ± 4.6 | 95.1 ± 3.1 | 0.289 |
| Total CHO (g/d) | 212.0 ± 61.9 | 218.0 ± 52.1 | 205.0 ± 72.8 | 0.611 |
| Total CHO/bw (g/kg/d) | 4.3 ± 1.3 | 4.5 ± 1.2 | 4.0 ± 1.5 | 0.371 |

*Data presented as mean ± SD. BMI, body mass index; BW, body weight; CHO, carbohydrates; CIQ, Control-IQ.*
